# The effect of physician’s recommendation on seasonal influenza immunization in children with chronic diseases

**DOI:** 10.1186/1471-2458-12-984

**Published:** 2012-11-15

**Authors:** Elisabetta Pandolfi, Maria Giulia Marino, Emanuela Carloni, Mariateresa Romano, Francesco Gesualdo, Piero Borgia, Roberto Carloni, Alfredo Guarino, Antonietta Giannattasio, Alberto E Tozzi

**Affiliations:** 1 Bambino Gesù Children’s Hospital, IRCCS, Piazza S. Onofrio 4, Rome 00165, Italy; 2 Department of Public Health, University of Tor Vergata, Rome, Italy; 3 Agency for Public Health, Rome, Lazio Region, Italy; 4 Health Regional Agency, Genova, Liguria Region, Italy; 5 Federico II University, Naples, Italy

**Keywords:** Influenza, Immunization, Chronic disease, Children, Physicians, Recommendations

## Abstract

**Background:**

Despite recommendations by Health Authorities, influenza immunization coverage remains low in children with chronic diseases. Different medical providers involved in the management of children with chronic conditions may affect the pattern of influenza vaccine recommendations and coverage. The likelihood of vaccination by type of provider in children with chronic conditions is poorly understood. Therefore, the objectives of this study were to analyze the pattern and the effect of recommendations for seasonal influenza immunization provided by different physician profiles to families of children with chronic diseases and to measure the frequency of immunization in the study population.

**Methods:**

We recruited children with chronic diseases aged 6 months–18 years who subsequently presented to specialty clinics for routine follow-up visits, during spring 2009, in three Italian Regions Families of children with chronic diseases were interviewed during routine visits at reference centers through a face-to-face interview. We analyzed the following immunization predictors: having received a recommendation toward influenza immunization by a health provider; child’s sex and age; mothers and fathers’ age; parental education and employment; underlying child’s disease; number of contacts with health providers in the previous year. Influenza immunization coverage was calculated as the proportion of children who received at least one dose of seasonal influenza vaccine in the previous season. We calculated prevalence ratios and we used a generalized linear model with Poisson family, log link and robust error variance to assess the effect of socio-demographic variables, underlying diseases, and recommendations provided by physicians on influenza immunization.

**Results:**

We enrolled 275 families of children with chronic diseases. Overall influenza coverage was 57.5%, with a low of 25% in children with neurological diseases and a high of 91.2% in those with cystic fibrosis. While 10.6% of children who did not receive any recommendation toward influenza immunization were immunized, among those who received a recommendation 87.5-94.7% did, depending on the health professional providing the recommendation. Receiving a recommendation by any provider is a strong predictor of immunization (PR = 8.5 95% CI 4.6;15.6) Most children received an immunization recommendation by a specialty (25.8%) or a family pediatrician (23.3%) and were immunized by a family pediatrician (58.7%) or a community vaccinator (55.2%).

**Conclusions:**

Receiving a specific recommendation by a physician is a strong determinant of being immunized against seasonal influenza in children with chronic diseases independently of other factors. Heterogeneity exists among children with different chronic diseases regarding influenza recommendation despite international guidelines. Increasing the frequency of appropriate recommendations toward influenza immunization by physicians is a single powerful intervention that may increase coverage in children with chronic conditions.

## Background

Human influenza infection is associated with a high risk of hospitalization for serious complications in children with chronic conditions [1,2]. Mechanisms that increase the risk of influenza complications in children with chronic diseases depend on the specific underlying disease. Children with neurological disorders show an increased risk of aspiration, and may experience respiratory failure associated with influenza infection [3,4]. Children affected by cystic fibrosis may show pulmonary exacerbations caused by the influenza virus [5,6]. Diabetic children are prone to metabolic failure during influenza infection [7]. HIV-infected children and adolescents, especially persons with a low CD4 cell count or AIDS, can experience more severe complications of seasonal influenza [8]. High rates of influenza-associated complications and death have been reported in children with Down syndrome [9].

Most European countries have implemented selective immunization programs for children with chronic diseases, while in the US influenza immunization is offered to all children [10].

Although influenza immunization is crucial in children with chronic medical conditions, as underscored in international recommendations [10], influenza immunization coverage in these patients remains low in many European countries, including Italy [11-14].

Factors that can limit immunization uptake in this population include difficulty in identifying at-risk children and poor awareness of specific recommendations by health professionals [15,16]. Additional reasons reported for insufficient immunization uptake in this group of children include false contraindications and, less frequently, a reactivation of the underlying disease [16,17].

It is also well recognized that multilevel interventions, including recommendations to vaccinate provided by physicians, represent a powerful factor favoring immunization [18,19]. On the other hand, determinants of influenza immunization in children affected with chronic diseases may differ from those of the general healthy population and have not been sufficiently studied.

In Italy, as in other European countries, influenza vaccine is recommended and offered free of charge to all children with chronic conditions [20]. Most Italian children receive immunizations in public vaccination centers, while some are vaccinated by family pediatricians in their office, or, when they are affected by a chronic disease, by specialty pediatricians in reference centers. Hence, influenza immunization of children affected with chronic diseases possibly depends on recommendations provided by different health providers such as family pediatricians, specialty pediatricians, and community vaccinators.

In a previous study conducted on families of children with chronic diseases we observed insufficient immunization coverage and a significant delay for both routine and recommended immunizations [17].

While it is clear that recommendation for influenza vaccination by a health care provider is a strong predictor of vaccination, the likelihood of vaccination by type of provider in children with chronic conditions is poorly understood. Therefore, the objectives of this study were to analyze the pattern and the effect of recommendations for seasonal influenza immunization provided by different physician profiles to families of children with chronic diseases and to measure the frequency of immunization in the study population.

## Methods

### Study design and sample selection

We recruited children affected with chronic diseases aged 6 months–18 years who subsequently presented to specialty clinics for routine follow-up visits, during spring 2009, in three Italian Regions. All enrolled patients were in the routine care of the specialty clinics. The population served by the three Regions is of approximately 13.000.000 people and represents nearly 22% of the Italian population overall. The three Regions were selected to represent three geographic areas of Italy (North, Center and South), in order to account for geographical trends. In the three Regions included in the study the number of children 6 months-18 years old affected with any chronic disease is approximately 120.000. The number of children affected with the chronic diseases considered in the study referring to the participating specialty clinics is nearly 5.000.

Eligibility criteria included signing an informed consent by parents and having one of the following conditions: type 1 diabetes, cystic fibrosis, Down syndrome, HIV infection, or a neurological disease including neurological conditions impairing the respiratory function (epilepsy or other diseases associated with seizures, neuromuscular disease, encephalopathy, genetic diseases with neurological impairment, hydrocephalus).

We chose to consider these conditions because they represent a wide spectrum of chronic diseases in terms of morbidity, mortality and pattern of contacts with different health providers, and because they experience frequent complications during influenza [4-9]. Moreover the selected study population includes children affected with diseases that may be perceived with different levels of severity: this may affect the frequency of recommendations and immunization coverage. The children’s diagnoses were confirmed by the reference centers in charge of following these patients. The study was approved by the ethical committee of the Bambino Gesù Children’s Hospital, Rome, Italy.

### Enrollment, data collection and statistical analysis

Families of children with chronic diseases were interviewed during routine visits at reference centers. One interviewer visited each specialty clinic once a week for three months during visit hours, and performed a face-to-face interview with parents of children affected with chronic diseases who attended the clinic. All the families of patients who presented during the day in which the interviewer was on duty at the Center were recruited. Since appointments for these visits were programmed, families were requested to bring proof of the child’s vaccinations in order to review and record the child’s vaccination history. Families that had not brought their child’s immunization card were not enrolled in the study. Interviews were conducted through a standardized questionnaire collecting information regarding the child's vaccination history, recommendation received by type of provider, and barriers encountered by parents to completing immunizations in a timely manner. Information on socio-demographic characteristics, type of underlying disease, and contacts with health providers in the previous year were also recorded.

Immunization coverage was calculated as the proportion of children who received at least one dose of seasonal influenza vaccine in the previous season on the total number of children. Confidence intervals of proportions were calculated as 95% interval. Differences in proportions were evaluated through the Chi square test whereas differences between means were analyzed through the ANOVA F test or the Kruskal-Wallis test. We calculated prevalence ratios and we used a generalized linear model with Poisson family, log link and robust error variance to assess the effect of socio-demographic variables, underlying diseases, and recommendations provided by physicians on influenza immunization [21]. We considered the following immunization predictors: child’s sex (reference: females) and age; parental age; parental education level (high school diploma or higher vs lower levels); parental employment status; child’s underlying disease (reference: neurological diseases); recommendation received by a health provider; number of visits by health provider in the last year. Number of visits was treated as a continuous variable. Independent variables included in the multivariate analysis were selected among those associated with influenza immunization with a p < 0.20 at the univariate analysis. Crude and adjusted prevalence ratios and their 95% confidence intervals were used as measures of effect. Software STATA 10 was used to analyze data.

A tree graph was used for describing the path that families of children with chronic diseases followed, from the moment they received the influenza vaccine recommendations to the administration of the vaccine by type of health provider.

## Results

We enrolled 275 children, whose socio-demographic characteristics are shown in Table 1.

**Table 1:** General characteristics of the study population

	**Cystic fibrosis**	**Type 1 Diabetes**	**Down Syndrome**	**HIV infection**	**Neurological disease**	**Total**	**p**	
	N = 57	N = 52	N = 70	N = 36	N = 60	N = 275		
Age, years; mean (SD)	9.5 (4.9)	10.3 (3.9)	5.5 (3.9)	10.4 (4.0)	8.8 (5.0)	8.6 (4.8)	<0.01	a
Females; N (%)	23 (40.4)	23 (44.2)	37 (52.9)	21 (58.3)	22 (36.7)	126 (45.8)	0.17	b
Foreigners; n (%)	2 (3.5)	0	2 (2.9)	4 (11.1)	0	8 (2.9)	0.02	b
Mother’s age, years; mean (SD)	39.2 (6.5)	41.8 (4.9)	40.9 (6.5)	40.1 (9.7)	38.8 (6.9)	40.1 (6.8)	0.11	c
Father’s age, years; mean (SD)	42.9 (6.4)	44.7 (5.3)	43.0 (6.9)	43.5 (7.9)	41.9 (6.6)	43.1 (6.6)	0.25	c
Mothers with at least high school diploma; N (%)	40 (70.2)	30 (57.7)	48 (68.6)	5 (20.0)	32 (54.2)	155 (58.9)	<0.01	b
Employed mothers; N (%)	23 (40.3)	33(63.5)	35 (50.0)	8 (30.8)	19 (31.7)	118 (44.5)	<0.01	b
Fathers with at least high school diploma; N (%)	32 (56.1)	26 (53.1)	43 (64.2)	3 (15.8)	33 (56.9)	137 (54.8)	<0.01	b
Employed fathers; N (%)	54 (94.7)	46 (92.0)	65 (95.6)	14 (70.0)	55 (94.8)	234 (92.5)	<0.01	b
Number of visits by specialty pediatrician in the last year; mean (SD)	4.5 (1.3)	4.5 (1.5)	2.9 (2.0)	5.9 (0.6)	3.9 (1.3)	4.1 (1.7)	<0.01	a
Number of visits by family pediatrician in the last year; mean (SD)	2.9 (2.3)	3.6 (2.0)	4.4 (1.7)	2.4 (2.3)	4.5 (1.7)	3.7 (2.1)	<0.01	a
Number of visits by community vaccinators in the last year; mean (SD)	0.6 (0.9)	0.3 (0.5)	0.8 (1.3)	0.3 (0.5)	0.5 (1.1)	0.5 [1]	0.26	a

Notes:

a) Kruskal-Wallis test.

b) Chi squared test.

c) Anova F-test.

Missing values range: 1–17.

None of the families eligible for the study declined participation. The mean age of participants was 8.6 years. A proportion of 46% were females. Mothers’ and fathers’ ages were similar across the groups with different conditions. Patients with HIV infection were more likely foreigners, had parents with a lower education level, and their fathers were more likely unemployed, compared with patients affected with other diseases. The average number of visits differed by health provider, being highest for specialty pediatricians, and lowest for community vaccinators, although children with Down syndrome and neurological diseases had frequent contacts with family pediatricians.

Children affected by cystic fibrosis and HIV infection were more frequently recommended to receive influenza immunization compared to patients with other chronic diseases (Table 2). Children with cystic fibrosis and HIV infection received influenza vaccination recommendations mainly from specialty pediatricians, while children affected with other diseases were more frequently recommended toward immunization by family pediatricians (Table 2). Fewer families reported receiving an influenza recommendation from community vaccinators. However it should be considered that contacts with specialty pediatricians and family pediatricians were frequent, while contacts with community vaccinators were rare.

**Table 2:** Frequency of recommendation toward influenza immunization by health provider and disease; proportion (95%CI)

**Recommendation provided by**	**Cystic fibrosis**	**Type 1 Diabetes**	**Down Syndrome**	**HIV infection**	**Neurological disease**	**Total**	**p**
	N = 57	N = 52	N = 70	N = 36	N = 60	N = 275	
Specialty pediatrician	27/57	7/52	3/70	31/36	3/60	71/275	<0,001
	47.4 (34.0 - 60.7)	13.5 (3.9 - 23.1)	4.3 (0.0 - 9.1)	86.1 (74.2 - 98.0)	5.0 (0.0 - 10.7)	25.82 (20.6 - 31.0)	
Family pediatrician	12/57	16/52	26/70	0/36	10/60	64/275	<0,001
	21.1 (10.1 - 32.0)	30.8 (17.8 - 43.7)	37.1 (25.5 - 48.7)	0.0 (0.0 - 0.0)	16.7 (7.0 - 26.4)	23.3 (18.2 - 28.3)	
Community vaccinators	3/57	1/52	3/70	0/36	1/60	8/275	<0,001
	5.3 (0.0 - 11.2)	1.9 (0.0 - 5.7)	4.3 (0.0 - 9.1)	0.0 (0.0 - 0.0)	1.7 (0.0 - 5.0)	2.9 (0.9 - 4.9)	
Two or more physicians	9/57	5/52	2/70	2/36	1/60	19/275	<0,001
	15.8 (6.0 - 25.6)	9.6 (1.3 - 17.9)	2.9 (0.0 - 6.9)	5.6 (0.0 - 13.4)	1.7 (0.0 - 5.0)	6.9 (3.9 - 9.9)	
No recommendation	6/57	23/52	36/70	3/36	45/60	113/275	<0,001
	10.5 (2.3 - 18.7)	44.2 (30.3 - 58.2)	51.4 (39.4 - 63.4)	8.3 (0.0 - 17.8)	75.0 (63.7 - 86.3)	41.1 (35.2 - 46.9)	

The overall influenza coverage was 57.5%, with a low of 25% in children with neurological diseases and a high of 91.2% in those affected with cystic fibrosis (Table 3). Children who did not receive a recommendation toward influenza immunization had the lowest coverage. Although the pattern of recommendations differed by disease group, recommendation toward influenza immunization resulted in a high rate in all categories.

**Table 3:** Immunization coverage by provider recommendation and disease; proportion

**Recommendation provided by**	**Cystic fibrosis**	**Type 1 Diabetes**	**Down Syndrome**	**HIV infection**	**Neurological disease**	**Total**	**p**
	**N = 57**	**N = 52**	**N = 70**	**N = 36**	**N = 60**	**N = 275**	
Specialty pediatrician	26/27 (96.3%)	7/7 (100%)	3/3 (100%)	24/31 (77.4%)	3/3 (100%)	63/71 (88.7%)	<0.001
Family pediatrician	11/12 (91.7%)	14/16 (87.5%)	24/26 (92.3%)	0	9/10 (90%)	58/64 (90.6%)	<0.001
Community vaccinators	3/3 (100%)	1/1 (100%)	2/3 (66.7%)	0	1/1 (100%)	7/8 (87.5%)	<0.001
Two or more physicians	9/9 (100%)	5/5 (100%)	2/2 (100%)	1/2 (50%)	1/1 (100%)	18/19 (94.7%)	<0.001
No recommendation	3/6 (50%)	3/23 (13%)	5/36 (13.9%)	0	1/45 (2.2%)	12/113 (10.6%)	<0.001
Total coverage	52/57 (91.2%)	30/52 (57.7%)	36/70 (51.4%)	25/36 (69.4%)	15/60 (25%)	158/275 (57.5%)	<0.001

**Figure 1:**
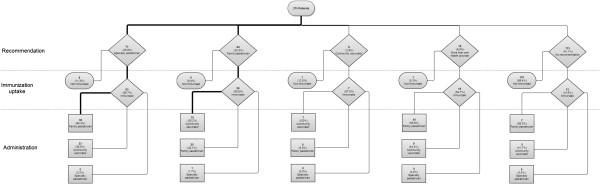
**Illustrates the path that children followed from immunization recommendation to immunization uptake.** Thicker lines represent the most frequent path for each of the resulting categories.

Since children included had different recommendation patterns, we reviewed their path from recommendation to receipt of influenza vaccine. We observed that the most frequent immunization path was receiving a recommendation to be immunized by a specialty or a family pediatrician and then being immunized by a family pediatrician or a community vaccinator (Figure 1). Specialty pediatricians rarely administered the influenza vaccine.

Since the effect of a recommendation received by a health provider may be confounded by several factors, we applied a multivariate analysis showing that receiving a recommendation by any physician is a strong predictor of being vaccinated against seasonal influenza independently of other variables (PR = 8.5 95% CI 4.6;15.5). In fact, children who received a specific recommendation to receive influenza immunization by one of the three providers considered were significantly more likely to be vaccinated than those who did not receive any recommendation (Table 4). The prevalence ratio of being immunized by recommendation of different health providers was homogeneous and did not differ if a child received a recommendation by more than one health provider. None of the other potential determinants of immunization included in the analysis was significantly associated with influenza uptake (Table 4).

**Table 4:** Determinants of influenza immunization; multivariate analysis

	**Univariate analysis**			**Multivariate analysis**		
**Determinant**	**PR (95% CI)**		**p**	**PR (95% CI)**		**p**
Child's age (continuous)	1.0	(0.98 - 1.03)	0.588			
Child male gender	0.9	(0.8 – 1.1)	0.523			
Mother's age (continuous)	1.0	(0.99 – 1.02)	0.448			
Father's age (continuous)	1.0	(0.99 – 1.03)	0.165	1.0	(0.99 – 1.0)	0.851
Mother with at least high school diploma	1.2	(1.0 – 1.5)	0.105	1.0	(0.9 – 1.2)	0.682
Father with at least high school diploma	1.0	(0.8 – 1.2)	0.815			
Mother employed	1.1	(0.9 – 1.4)	0.292			
Father employed	1.1	(0.7 – 1.7)	0.708			
Neurological diseases (reference)						
Diabetes	2.3	(1.4 – 3.8)	0.001	1.3	(1.0 – 1.8)	0.099
Cystic fibrosis	3.6	(2.3 – 5.7)	<0.001	1.3	(1.0 – 1.8)	0.080
HIV infection	2.8	(1.7 – 4.5)	<0.001	1.0	(0.7 – 1.5)	0.837
Down syndrome	2.1	(1.3 – 3.4)	0.004			
No recommendation (reference)						
Recommendation by specialty pediatrician	8.4	(4.9 – 14.4)	<0.001	8.4	(4.5 – 15.7)	<0.001
Recommendation by family pediatrician	8.5	(5.0 – 14.7)	<0.001	8.6	(4.7 – 15,6)	<0.001
Recommendation by community vaccinator	8.2	(4.5 – 15.0)	<0.001	7.9	(4.1 – 15.0)	<0.001
Recommendation by two or more physicians	8.9	(5.2 – 15.4)	<0.001	8.8	(4.7 – 16.2)	<0.001
Number of visits by specialty pediatrician (continuous)	1.1	(1.0 – 1.1)	0.032	1.0	(0.95 – 1.04)	0.940
Number of visits by family pediatrician (continuous)	0.9	(0.9 – 1.0)	0.011	1.0	(0.9 – 1.0)	0.109
Number of visits by community vaccinator (continuous)	0.9	(0.8 – 1.0)	0.034	1.0	(0.9 – 1.0)	0.273

Due to missing values, the total number of subjects included in the multivariate model is 250.

PR = Prevalence Ratio.

## Discussion

Our study shows that receiving a specific recommendation for influenza immunization by any physician is a single strong determinant of influenza immunization uptake in children with chronic diseases. This positive influence is independent from socio-demographic characteristics, number of contacts with health providers, and underlying disease.

We found that immunization coverage in children with chronic conditions substantially changes depending on the underlying disease, as already observed [22], despite all participants in the study belonged to categories addressed by international guidelines on influenza immunization [23]. Patients with neurological disorders had the lowest immunization coverage and they were those with the lowest recommendation level. This finding might be explained by different perceptions of influenza infection severity in different chronic conditions by health professionals, as observed for neurological disorders and type 1 diabetes in other studies [24,25].

Many studies focused on barriers to immunization and on multilevel interventions to increase vaccination coverage in healthy and chronic patients [26]. Strong scientific evidence exists showing that multicomponent interventions including education are effective in improving immunization coverage [19]. Physicians willingness and capabilities to recommend vaccinations are affected by the perceived relevance of the vaccines, by knowledge on their contraindications, by the health care system organization, and by communication between providers [26,27]. Also non-integrated interventions may be effective in improving immunization coverage in children with chronic conditions, but the evidence is weak [19]. Multicomponent interventions including reminder systems or other educational initiatives were not in place in our setting. Increasing the ability to provide a recommendation toward immunization by physicians may represent therefore a simple and efficacious strategy in settings where multicomponent interventions are not in place.

Different health providers are involved in the management of children with chronic conditions and multiple contacts with different specialists may result in different paths from recommendation to vaccine administration. We focused on the health providers that most frequently are in contact with these children in the Italian setting and whose recommendation may influence their decision to receive influenza immunization. We found that having received a recommendation by any health provider is associated with an increased likelihood of receiving influenza immunization. Since health professionals included in our analysis may provide recommendation and administer influenza vaccines as well, we investigated the path from recommendation to vaccination administration that these children followed. Although the number of visits by a certain provider is not associated with immunization uptake, families of patients with chronic diseases refer most frequently to specialty and family pediatricians. Given that recommendation to immunize is highly effective, increasing the frequency of recommendation by health providers may substantially change immunization uptake.

This study had several strengths and limitations. We did not select a sample of clinics in each Region but we rather selected the largest Reference Centers for the chronic diseases considered in the study. Although our study was conducted on a heterogeneous population of children with different chronic diseases whose information on immunization was carefully reviewed, it is likely that we selected families with a high level of attention to their child’s disease. It must be underlined that the follow up for chronic diseases is entirely free of charge in the Italian health care system and usually large reference centres concentrate the largest population of children with chronic diseases in their catchment area. Moreover, it is of note that families frequently reported having received a recommendation to immunize against influenza by different health providers, and that the effect of these recommendations is strong as well. Although we allowed participation in the study only to families who brought in their immunization card, we do not feel that this may have led to a significant bias since their number was negligible. Patients in charge to reference centers enrolled in the study were strictly monitored for at least six months and they were reminded to bring in their immunization card at each appointment.

We included in the study only a group of chronic disease that we believe representative but we excluded some common diseases such as asthma. While asthma is the most common chronic condition in childhood for which influenza vaccine is recommended, we felt it would deserve a specific study to investigate determinants of influenza immunization.

Finally, we might have interviewed, in each health care centers, patients routinely visited by a single physician, whose behavior may not reflect the behavior of the entire team of physicians in the same clinic. However, the specialty clinics included in the study include a large number of physicians on duty, with a high turnover; moreover, we performed our weekly interviews in different weekdays, reducing the probability of meeting patients referring to the same physician.

Since information on recommendation was collected through interviews, this may be subject to recall bias and those who received influenza immunization may better recall having received a recommendation. However, although reporting bias is probable, it is unlikely that such a strong association is explained by these biases, and, therefore we believe that medical recommendations are essential to improve immunization coverage.

Finally, we cannot say if our results are applicable to other countries and settings. However, since the number of health providers in contact with families of children with chronic diseases is high, it may well be that recommendation toward influenza immunization may be provided at multiple levels in other settings. Hence, similar studies should be conducted in other settings for confirming the results.

## Conclusions

Our study suggests that increasing the frequency of recommendations to receive influenza immunization by health professionals most frequently in contact with children with chronic diseases is a single intervention that may significantly increase influenza immunization coverage in settings where other multilevel interventions to favor immunization are not in place. Public health actions including education of different health care providers and improvement of communication to patients should be pursued to achieve a higher influenza vaccine coverage in children at risk, as well to enhance the interventions.

## Competing interests

The authors declare that they have no competing interests.

## Authors’ contributions

EP coordinated the study and participated in the writing process and in the data review. M.G.M designed the study and drafted the manuscript. EC performed the statistical analysis. MR conducted the interviews, FG conducted the interviews and revised the final version of the manuscript. PB, RC, AG and AG conducted families’ interviews at each regional centre. AET conceived the study, participated in its design and coordination and drafted the manuscript. All authors read and approved the final manuscript.

## Pre-publication history

The pre-publication history for this paper can be accessed here:

http://www.biomedcentral.com/1471-2458/12/984/prepub
